# Novel Angiotensin-Converting Enzyme Inhibitory Peptides Identified from Walnut Glutelin-1 Hydrolysates: Molecular Interaction, Stability, and Antihypertensive Effects

**DOI:** 10.3390/nu14010151

**Published:** 2021-12-29

**Authors:** Jing Wang, Guoliang Wang, Yufeng Zhang, Runguang Zhang, Youlin Zhang

**Affiliations:** 1 College of Food Engineering and Nutritional Science, Shaanxi Normal University, Xi’an 710119, China; wj_sxxqsfxy@163.com (J.W.); guoliangw@snnu.edu.cn (G.W.); zhangyufeng062@163.com (Y.Z.); sunshine@snnu.edu.cn (R.Z.); 2 College of Life Sciences and Food Engineering, Shaanxi Xueqian Normal University, Xi’an 710100, China; 3 The Key Laboratory of Se-Enriched Products Development and Quality Control, Ministry of Agriculture, Ankang 725000, China; 4 Hainan Engineering Center of Coconut Further Processing, Coconut Research Institute of Chinese Academy of Tropical Agricultural Sciences, Wenchang 571339, China

**Keywords:** walnut glutelin-1, ACE inhibitory peptides, purification, molecular docking, antihypertensive effect

## Abstract

In recent years, angiotensin-converting enzyme (ACE) inhibitory peptide has become a research hotspot because of its essential role in maintaining human blood pressure balance. In this study, two novel ACE inhibitory peptides of Val-Glu-Arg-Gly-Arg-Arg-lle-Thr-Ser-Val (Valine-Glutamate-Arginine-Glycine-Arginine-Arginine-Isoleucine-Threonine-Serine-Valine, VERGRRITSV) and Phe-Val-Ile-Glu-Pro-Asn-Ile-Thr-Pro-Ala (Phenylalanine-Valine-Isoleucine-Glutamate-Proline-Asparagine-Isoleucine-Threonine-Proline-Alanine, FVIEPNITPA) were isolated and purified from defatted walnut meal hydrolysates through a series of preparation processes including ultrafiltration, Sephadex G-15 gel chromatography, and reverse high performance liquid chromatography (RP-HPLC). Both peptides showed high ACE inhibitory activities. The molecular docking study revealed that VERGRRITSV and FVIEPNITPA were primarily attributed to the formation of strong hydrogen bonds with the active pockets of ACE. The binding free energies of VERGRRITSV and FVIEPNITPA with ACE were −14.99 and −14.69 kcal/mol, respectively. Moreover, these ACE inhibitory peptides showed good stability against gastrointestinal enzymes digestion and common food processing conditions (e.g., temperature and pH, sugar, and salt treatments). Furthermore, animal experiment results indicated that the administration of VERGRRITSV or FVIEPNITPA exhibited antihypertensive effects in spontaneously hypertensive rats. Our results demonstrated that walnut could be a potential source of bioactive peptides with ACE inhibitory activity.

## 1. Introduction

Walnut (*Juglans regia* L.) is known as one of the world’s famous “four dried fruits” together with almonds, cashews, and hazelnuts. It has a long history of cultivation and a large planting area in China. At present, edible oil extraction is the main processing method for walnut kernels.

Walnut meal is an essential by product in the walnut processing industry, and it contains a variety of nutrients, is rich in protein (40–50%) and essential amino acids [1]. However, walnut meal and related products have limited market acceptance due to their bitter and astringent taste, so most are simply processed and used as fertilizers, or even directly discarded as waste. This processing method wastes high-quality protein resources and pollutes the natural environment [2]. Therefore, making full use of walnut protein and developing high value-added products is of great significance to improve the level of deep processing and comprehensive utilization of this raw material.

Hypertension is a significant risk factor for heart diseases, stroke, arteriosclerosis, and other diseases. This condition threatens the health of approximately 20% of adults worldwide [3,4]. Studies have shown that angiotensin–converting enzyme (ACE) is a central factor of the renin–angiotensin system and kallikrein–kinin system, and plays a crucial role in increasing blood pressure (BP) [5,6]. Therefore, inhibiting the ACE’s activity has become one of the most effective methods to control blood pressure. Several antihypertensive drugs are designed based on the mechanism of inhibiting ACE activity. Many chemically synthesized ACE inhibitors such as captopril, lisinopril, and enalapril, have been widely used to control hypertension. Nevertheless, previous reports show that the long-term use of these drugs usually has serious side effects, such as cough, hyperkalemia, skin rashes, kidney injury, and taste disorders [7,8]. Some antihypertensive peptides, derived from natural food, are considered safe for consumption, and their antihypertensive effects are as effective as synthetic drugs but with less or no adverse side effects compared with synthetic ACE inhibitor drugs (as described above) [9,10,11,12]. Therefore, more and more food-derived antihypertensive peptides have been researched and reported.

In general, ACE inhibitory peptides directly bind or indirectly induce conformational changes at enzyme active sites [13]. The molecular interaction between the purified peptide and ACE is essential to determine the inhibitory activity [14]. Therefore, it is important to identify natural ACE inhibitory peptides from different species and study the interaction with ACE molecules.

At present, the preparation methods of food-derived antihypertensive peptides primarily include microbial fermentation, enzymatic hydrolysis, and gene synthesis [15,16,17], among which, enzymatic hydrolysis is the most widely used. Although ACE inhibitory peptides derived from walnut protein have been reported in the literature [18,19], no research has been conducted on ACE peptides isolated and purified from the component protein (glutelin-1 hydrolysate). In addition, previous studies primarily focused on the separation, purification, and identification of walnut ACE inhibitory peptides. Very few reports have focused on the mechanism, the effects of lowering blood pressure in rats, the stability of the simulated gastrointestinal (GI) environment, and typical food processing conditions.

For this study, we have prepared glutelin-1 from a walnut meal. After being hydrolyzed by pepsin, the glutelin-1 hydrolysate was separated and purified by membrane separation, gel chromatography, and high-performance liquid chromatography (HPLC–MS/MS). Finally, highly active ACE inhibitory peptides could be obtained. The amino acid sequences of the ACE inhibitory peptides were identified through N-terminal sequence analysis. Moreover, we evaluated the antihypertensive effects of walnut glutelin-1 ACE inhibitory peptides in vivo using a spontaneously hypertensive rat (SHR) model. The ACE inhibitory mechanism of the peptide was explored through molecular docking simulation. We have also investigated the stability of walnut glutelin-1 ACE inhibitory peptides under different food processing conditions and simulated GI digestion environments. The outcomes of this study can improve the deep processing and comprehensive utilization of walnuts and provide theoretical and technical references for the research and development of walnut antihypertensive peptides.

## 2. Materials and Methods

### 2.1. Materials

The Grain, Oil and Protein Engineering Laboratory of Shaanxi Normal University (Xi’an, China) provided the defatted walnut meal. ACE (from rabbit lung) and hippuryl-histidyl-leucine (HHL) were purchased from Sigma Co., St. Louis, MO, USA. Pepsin (3 × 10^3^ U/g) was obtained from Solarbio Science & Technology, Co., Ltd. (Beijing, China). High-performance liquid chromatography grade acetonitrile and trifluoroacetic acid were purchased from Fischer Scientific Co. (Waltham, MA, USA). The enzyme-linked immunosorbent assay kit for the detection of ACE, angiotensinogen (AGT), aldosterone (ALD), and Endothelin-1(ET-1) was purchased from Jiangsu Jingmei Biotechnology Co., Ltd. (YanCheng, China). The enzyme-linked immunosorbent assay kit for the detection of Ang II was obtained from Shanghai Kexing Biotechnology Co., Ltd., (Shanghai, China). and the nitric oxide (NO) detection kit was a product of Nanjing Jiancheng Institute of Biological Engineering (Nanjing, China), and other chemical reagents used in the experiment were of analytical grade.

### 2.2. Preparation of Glutelin-1 in Degreased Walnut Meal

The glutelin-1 fractions were prepared according to our previously published method [20]. In short, the walnut meal was degreased using n-hexane (1:10, g/mL) for 2 h, then dried, ground, and passed through a mesh sieve. Next, the continuous extraction method was used to extract albumin, globulin, prolamin, glutelin-1, and glutelin-2 in walnut meal with deionized water, 0.4 M NaCl, 70% (*v*/*v*) ethanol, 50% (*v*/*v*) glacial acetic acid, and 0.1 M NaOH. After centrifugation, each extract went through dialysis and lyophilization, the final power obtained was stored at −20 °C until use.

### 2.3. Preparation of Glutelin-1 Hydrolysate

We prepared glutelin-1 hydrolysate according to our previous method [20]. In short, the pepsin and glutelin-1 solution were mixed at a ratio of 4:100 (*w*/*v*). The pH of the mixture was adjusted to 1.65 with 1 M HCl and enzymatically hydrolyzed at 45 °C for 6 h. The reaction was terminated by heating the solution in a 95 °C water bath for 10 min. Subsequently, the enzymatic hydrolysate was centrifuged at 10,000 rpm/min for 20 min at 4 °C, and the supernatant was freeze-dried and stored at −20 °C for further analysis.

### 2.4. Separation and Purification of the ACE-Inhibitory Peptides from Glutelin-1 Hydrolysate

An ultrafiltration unit (Pellicon XL, Millipore, Billerica, MA, USA) was used to obtain three fractions named A1 with molecular weight (MW) > 5000 Da, A2 with MW 3000–5000 Da, and A3 with MW < 3000 Da from the glutelin-1 hydrolysate by an ultrafiltration unit. The fraction with the highest ACE inhibitory activity was sequentially isolated and purified by a Sephadex G-15 gel column (2.6 cm × 100 cm, Shanghai Huxi Analytical Instrument Factory Co., Ltd., Shanghai, China) and eluted with distilled water at a flow rate of 0.6 mL/min. Its loading concentration was 100 mg/mL, and the absorbance was measured at 280 nm. Then, with specific conditions, a reversed-phase high-performance liquid chromatography (RP-HPLC) equilibrated with a C18 column (4.5 mm × 250 mm, Shimadzu Corporation LC-20A, Kyoto, Japan). First, two eluents consisting of solvent A (in distilled water with 0.1% (*v*/*v*) TFA) and solvent B (acetonitrile with 0.1% (*v*/*v*) trifluoroacetate) were eluted linearly at a flow rate of 1.0 mL/min for 30 min. The monitoring wavelengths at this stage were 220 nm and 280 nm, respectively. All fractions’ ACE inhibitory activity and peptide content could be determined after being collected and lyophilized.

### 2.5. Amino Acid Sequence Analysis of the Purified Peptides

The amino acid sequences and molecular mass of the purified peptides were determined by Edman degradation using an automatic protein polypeptide sequencer (Shimadzu Corporation, PPSQ-30A, Kyoto, Japan) and a mass spectrometer (Shimadzu Corporation, LC-20AD XR, Kyoto, Japan).

### 2.6. Commercially Synthesis of the Identified Peptides

The synthetic peptides were obtained by a solid phase procedure (KMD Bioscience, TianJin, China) according to the previously known amino acid sequence. They were purified by RP-HPLC-MS/MS (purity > 95%) and stored at −20 °C for further analysis.

### 2.7. Determination of ACE Inhibitory Activity

Following a previous method reported by Zheng et al. (2017) [21], we could determine the ACE inhibitory activity of glutelin-1 hydrolysate, separated fractions, active peptides, and all the other samples with 1.0 mg/mL Captopril as a positive control. The IC_50_ value (the concentrationof the inhibitory peptide (μM) had to inhibit 50% of the ACE activity) was calculated by linear regression of the curve.

The following formula was used to calculate the ACE inhibition rate (%):

$$\mathrm{ACE}\;\mathrm{inhibition}\;\mathrm{rate}\left(\%\right)=(1-\frac{\mathrm{As}}{\mathrm{Ac}})\times 100$$

where Ac represents ACE solution’s absorbance without an inhibitor and As represents the mixture containing samples.

### 2.8. Molecular Docking

The RCSB Protein Data Bank (https://www.rcsb.org, accessed on 2 February 2021) was used to download the crystal structure of the ACE (Protein Data Bank identification (PDB ID): 1O86, Resolution: 2.00 Å). The peptide structure was processed on the molecular manipulation environment platform (Molecular operating environment (MOE) 2019.1), including steps such as removing water and ions, protonation, addition of missing atoms, and completion of missing groups. Moe|Dock Module was used, and Amber10 was selected as the force field. One hundred conformations were generated after docking, and we selected the most negative conformations using the London DG scoring function. Finally, molecular visualization was processed by Pymol 2.3 software (Schrodinger Inc, NY, New York, USA).

### 2.9. Antihypertensive Effect in SHR

#### 2.9.1. BP Measurement

Thirty 10-week-old male spontaneously hypertensive rats (SHRs) and five normal male rats of the same age were used for the experiment. The rats were placed at 25 °C ± 1 °C, relative humidity of 45–55% and a light/dark cycle for 12 h. SHRs were randomly divided into 6 groups after one week of adaptation, while rats in the normal group (NG) were used as controls. The administration was continued for 28 days.

Rats in the normal and SHR groups were given 1.0 mL of 0.9% saline per day, while the positive control group was given captopril (10 mg/kg body weight) per day. The ACE inhibitory peptides treatment groups’ low and high doses were fed orally with 5 and 15 mg/kg (based on their body weight) by dissolving the peptides in 1.0 mL of 0.9% normal saline. The rats’ BP was measured by the tail-cuff method. The systolic blood pressure (SBP), diastolic blood pressure (DBP), and heart rate of all rats were measured after a single oral administration (0–8 h) and weekly (0–4 weeks). All measurements were repeated at least three times so the average value could be calculated.

#### 2.9.2. Assay for Serum ET-1, NO, ACE, Ang II, AGT, and ALD in SHR Rats

The upper serum samples were obtained by centrifugation at 3000× *g* at 4 °C for 10 min after the blood was taken from the abdominal aorta of all rats. The samples were stored at −20 °C, and the serum expression was detected using the ELISA kit (double antibody sandwich enzyme-linked immunosorbent assay).

### 2.10. Stability of the Synthetic Peptides

#### 2.10.1. GI Digestion Stability Analysis of the Synthesized Peptides

We conducted a GI digestion stability analysis of the synthesized peptides according to the method proposed by Khueychai et al. (2018) [5] with slight modifications. Briefly, the peptide solution was incubated with 1 mg/mL of pepsin solution (0.1 M HCl, pH 2.0) at 37 °C for 3 h. Subsequently, the pH value of the mixed solution was adjusted to 8.0 with 1.0 M NaOH, and 1.0 mg/mL of trypsin added and incubated at 37 °C for 4 h. Finally, the ACE inhibitory activity of the reaction solution could be determined after boiling for 10 min, cooling to room temperature, and centrifuging for 15 min at 10,000× *g*.

#### 2.10.2. Temperature, pH, Metal Ion, Sugar, and Salt Treatments Stabilities of the Synthesized Peptides

The effect of temperature, pH, metal ion, sugar, and salt treatments on the stabilities of two synthesized peptides, were determined by incubating 1.0 mg/mL of VERGRRITSV and FVIEPNITPA at 20 °C, 40 °C, 60 °C, 80 °C, and 100 °C for 2 h; 37 °C for 2 h at pH 2, 4, 6, 8, and 10; with 100 μg/mL of KCl, CaCl_2_, ZnSO_4_, MgSO_4_, and FeSO_4_ solution at 37 °C for 2 h; with glucose solutions of 2%, 4%, 8%, and 12% at 37 °C for 30 min; with NaCl solutions of 2%, 4%, 8%, and 12% at 37 °C for 30 min, respectively. The ACE inhibition rate was measured immediately after incubation.

### 2.11. Statistical Analyses

Each experiment had to be repeated at least three times, and the results were expressed as mean ± SD. One-way analysis of variance (ANOVA) was used to analyze the significant difference between the groups (*p* < 0.05). GraphPad Prism 6.0 (San Diego, CA, USA) and Microsoft Office programs, including Word and Excel, were used for statistical analysis and pictures drawing.

## 3. Results and Discussion

### 3.1. Isolation and Purification of ACE Inhibitory Peptides of Glutelin-1 Hydrolysate

**Figure 1:**
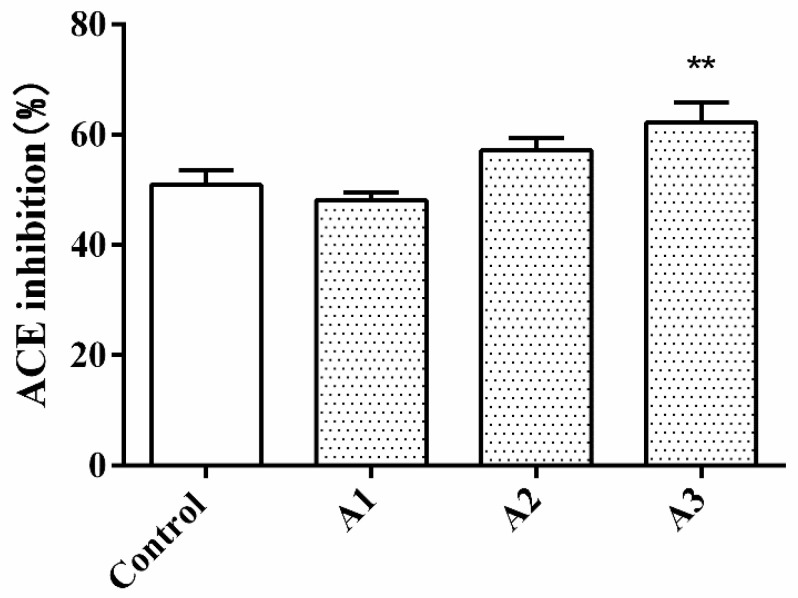
Angiotensin–converting enzyme (ACE) inhibitory rates of ultrafiltration fractions of glutelin-1 hydrolysates of walnut at the concentration of 1.0 mg/mL. Control: Glutelin-1 hydrolysate before separation and purification. All data are presented as the mean ± SD of triplicate results. ** *p* < 0.01, vs. the control, A1–A3 represent the three fractions obtained after ultrafiltration separation, A1 (MW > 5 kDa), A2 (3 < MW <5 kDa) and A3 (MW <3 kDa).

As shown in Figure 1, glutelin-1 hydrolysate was divided into three fractions through ultrafiltration membranes with molecular weight cut-offs of 5 and 3 kDa. The fractions were labeled as A1 (MW > 5 kDa), A2 (3 < MW < 5 kDa), and A3 (MW < 3 kDa). Among the three fractions, A3 (MW < 3 kDa) showed the highest ACE inhibitory activity (62.25% ± 1.54%), which was significantly higher than the glutelin-1 hydrolysate before ultrafiltration separation (50.91% ± 2.65%, *p* < 0.01).

Many studies have found a specific correlation between the molecular weight of peptides and ACE inhibitory activity. Low molecular weight (less than 3 kDa) peptides often had higher ACE inhibitory activity [22]. Our research results also indicated that the ACE inhibitory activity might be related to the size of the peptide.

**Figure 2:**
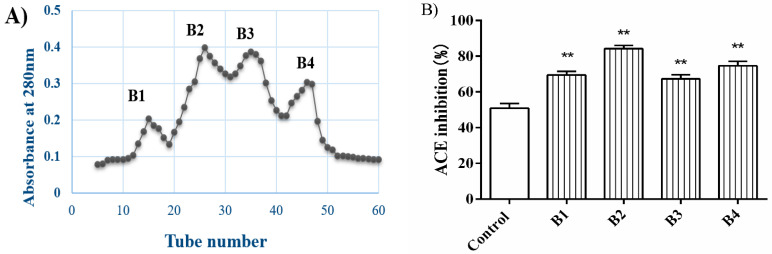
Sephadex G-15 gel chromatography separation of A3 (**A**) and ACE inhibitory rates of subfractions from A3 at the concentration of 1.0 mg/mL (**B**). Control: Glutelin-1 hydrolysate before separation and purification. All data are presented as the mean ± SD of triplicate results. ** *p* < 0.01, vs. the control, B1–B4 represent the four fractions separated by Sephadex G-15 gel chromatography.

**Figure 3:**
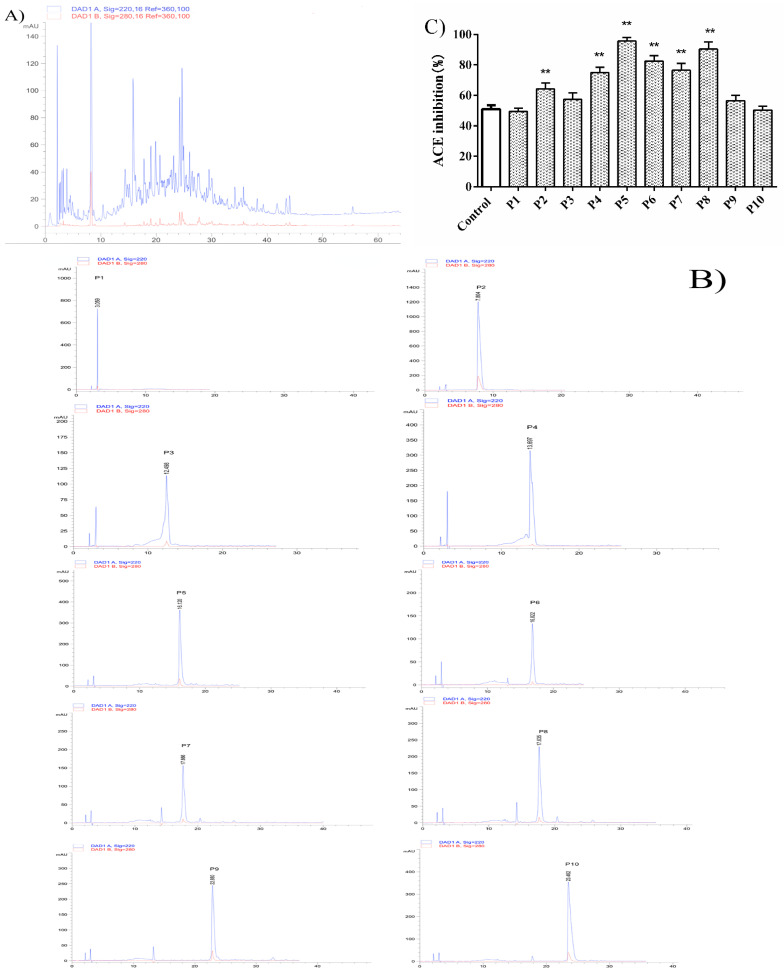
Chromatography of fraction B2 separated by RP-HPLC (Reverse high performance liquid chromatography) on a Shimadzu C18 column (5 μm, 4.6 × 250 mm) using a linear gradient of acetonitrile (20–100% in 0–25 min) in 0.1% TFA at a flow rate of 1.0 mL/min (**A**). Fractions P1-P10 (**B**). ACE inhibition activity of fractions P1-P10 (**C**) were separately isolated by analytical RP-HPLC. Control: Glutelin-1 hydrolysate before separation and purification. ** *p* < 0.01, vs. the control. P1–P10 represent 10 major fractions obtained after separation by RP-HPLC.

The A3 fraction was divided into four main fractions by Sephadex G-15 gel chromatography (Figure 2A). The ACE inhibitory activity of these four subfractions was significantly higher than that of the glutelin-1 hydrolysate (*p* < 0.01) (Figure 2B). In addition, fraction B2 showed higher ACE inhibitory activity (84.22% ± 1.84%) compared to the others, fraction B2 was again separated by RP-HPLC (Figure 3A). Ten major fractions labeled as P1–P10 were collected (Figure 3B). P5 and P8 displayed the highest ACE inhibitory activity (Figure 3C). RP-HPLC further purified these two fractions in an analytical C18 column, and later amino acid sequencing was performed.

### 3.2. Peptide Identification and Synthesis

The amino acid sequences of peptides P5 and P8 that showed the highest ACE inhibitory activity peptides, were analyzed using the Edman degradation method. As shown in Table 1, P5 was identified as Val-Glu-Arg-Gly-Arg-Arg-lle-Thr-Ser-Val (VERGRRITSV) with a molecular weight of 1172.35 Da. P8 was identified as Phe-Val-Ile-Glu-Pro-Asn-Ile-Thr-Pro-Ala (FVIEPNITPA) with a molecular weight of 1100.28 Da (Table 1). The two peptides were identified from walnut protein hydrolysate, and we did not find previous reports regarding this phenomenon. According to amino acid sequence results, the two peptides (VERGRRITSV and FVIEPDITPA) were synthesized via a solid-phase process to evaluate the ACE inhibitory activity. The ACE inhibitory peptides identified in this study had vigorous activity. VERGRRITSV and FVIEPNITPA displayed IC_50_ values of 6.82 μM and 6.36 μM, respectively.

Previous studies have evidenced that the ACE inhibitory activity of peptides is primarily related to molecular weight, hydrophobicity, amino acid composition, and peptide sequence [23]. Most of the reported competitive ACE inhibitory peptides usually have a molecular weight of less than 3 kDa and contain 2–12 amino acids. These short peptides interact with the ACE protein by competing for the active site of action [24]. The two peptides reported in this paper were similar. In addition, many studies found that the amino acid sequence of the peptide correlated with the ability to inhibit ACE activity. Many studies have shown that ACE inhibitory peptides usually have hydrophobic amino acid residues at the C-terminal or N-terminal sites [8,19]. When the N-terminal contains a hydrophobic amino acid, such as valine (V), phenylalanine (F), leucine (L), isoleucine (I), or a basic amino acid, the ACE activity is generally high. The higher ACE inhibitory activity of the pure peptide FVIEPNITPA might be related to its N-terminal containing 3 hydrophobic amino acids. Many peptides with high ACE activity contain valine at the N-terminal, such as VKPLPQSG, VAMPF, VPP, and VIIF [23,25,26]. Some antihypertensive peptides containing proline (P), valine (V), or phenylalanine (F) at the C-terminal sites showed high ACE inhibitory activity. Our results also support this idea. The C-terminal and N-terminal hydrophobic amino acids were indeed involved in the expression of ACE inhibitory activity. The high ACE inhibitory activity of VERGRRITSV identified in this study may be related to the N-terminal and C-terminal amino acids (Val). However, the ACE inhibitory peptides’ structure–activity relationship was not analyzed thoroughly.

### 3.3. Molecular Docking Simulation

**Figure 4:**
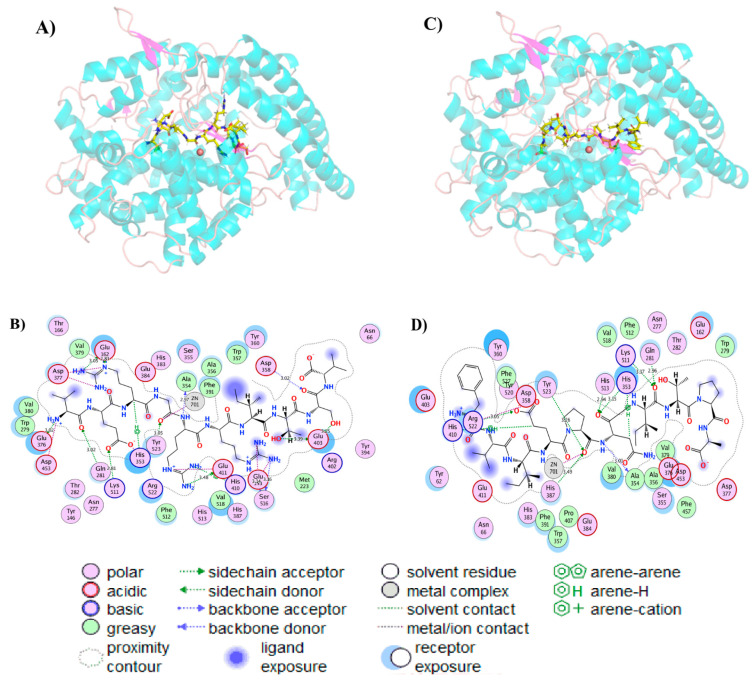
The best binding conformation of the pure peptides with ACE (PDB: 1O8A). 3D diagram of VERGRRITSV (**A**), 2D diagram of VERGRRITSV (**B**), 3D diagram of FVIEPDITPA (**C**), 2D diagram of FVIEPDITPA (**D**).

The interactions between the two purified bioactive peptides (VERGRRITSV and FVIEPNITPA) and ACE were characterized by molecular docking technology. As an important thermodynamic property, binding free energy is usually calculated with a biological system model [27]. As it can be inferred from the results of the lowest free energy between ACE and the two bioactive peptides shown in the molecular simulation docking model (Figure 4), both peptides could interact with ACE to form an ACE–ligand complex. The lower binding free energies (−14.99 and −14.69 kcal/mol for VERGRRITSV and FVIEPNITPA, respectively) indicated a high binding affinity between the peptides and the molecular ACE, which might be due to the existence of a large number of hydrogen bonds, hydrophobicity, electrostatic force and van der Waals. This possibility is valid, especially for the hydrogen bond, since it has been widely proved that it plays a vital role in maintaining the stability of ligand complexes [13,28]. For instance, multiple hydrogen bond interactions between peptides and ACE can increase the inhibition activity on ACE by stabilizing the structure of the non-catalytic enzyme–peptide complex [29]. In addition, the number of hydrogen bonds was often positively correlated with the interaction between peptides and ACE [14]. VERGRRITSV formed nine hydrogen bonds with residues Lys511, Gln281, Glu376, Glu162, Ala354, Tyr523, Glu411, ser516, and Glu403 and eight salt bridges with residues Glu376, His353, Asp377, His387, Glu411, Glu143, ser516, and Zn701 in ACE (Figure 4B). FVIEPNITPA established seven hydrogen bonds with residues Asn374, Glu376, Glu162, Ala354, Ala356, ser516, and Glu143 and two salt bridges with Zn701 and Arg522 in ACE (Figure 4D). It has previously been demonstrated that S1 with Tyr523, Glu384, and Ala354 residues, S2 with Tyr520, His513, Lys511, His353, and Gln281 residues, and S1 with Glu162 residue are the three main active site pockets of ACE [23,30]. Molecular docking results showed that the two bioactive peptides had different binding sites with ACE. VERGRRITSV could bind to ACE through S1, S2, and S1 pockets by the Ala354 and Tyr523 residues, Gln281, His353, and Lys511 residues, and Glu162 residue, respectively. FVIEPNITPA interacted with ACE though two pockets of S1 in Ala354 residue and S1 in Glu162 residue. These outcomes indicate that the strong interaction between VERGRRITSV and ACE may lead to great inhibitory activity. The ACE–ligand complex formed by docking was more stable than FVIEPNITPA. Besides, ACE’s active site was usually tetrahedral, which resulted from the coordination of zinc ions (Zn^2^) with Glu411, His387, and His383 [31]. VERGRRITSV and FVIEPNITPA might also compete with ACE to bind zinc ions, thereby destroying ACE’s active site and improving ACE inhibitory activity.

### 3.4. Antihypertensive Effect in SHR

#### 3.4.1. Effect of Oral Administration of Glutelin-1 ACE Inhibitory Peptide on SBP and DBP in SHR

**Figure 5:**
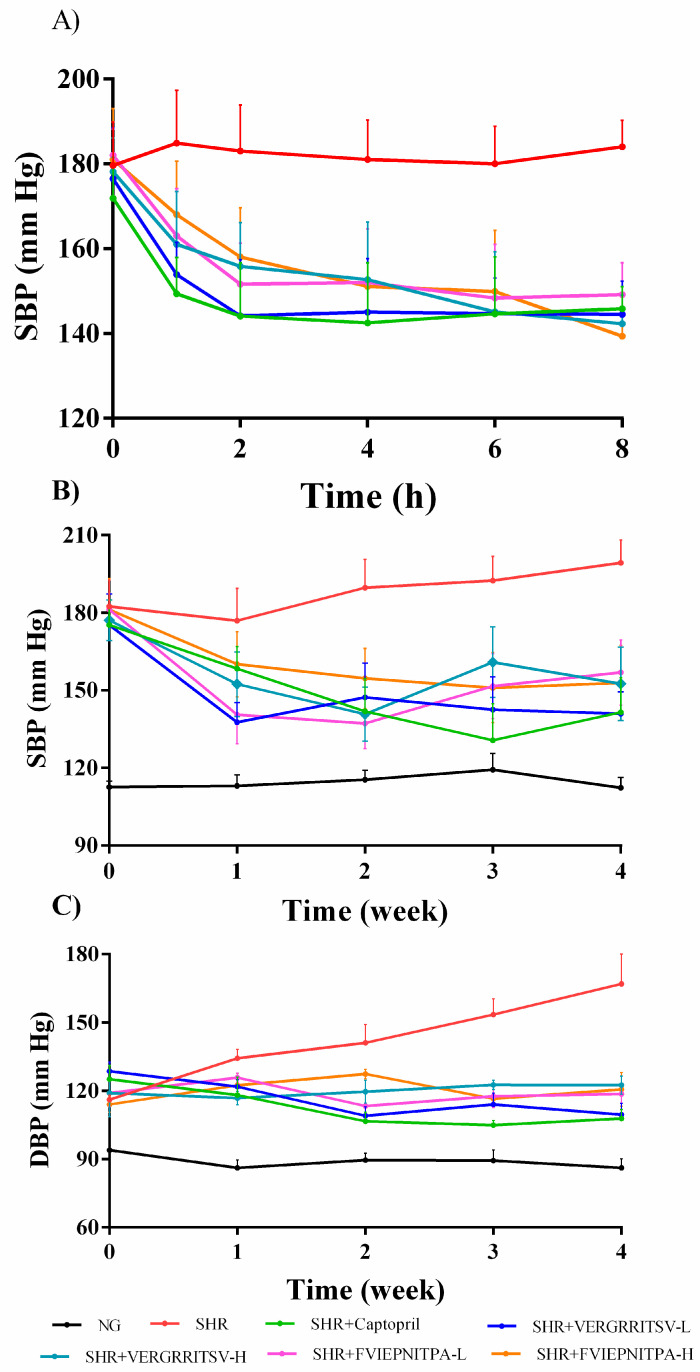
Effect of single oral administration (0–8 h) on SBP in SHRs (**A**); four weeks oral administration on SBP (**B**), and DBP in SHRs (**C**). NG represents rats in the normal group.

It has previously been reported that some ACE inhibitory peptides have good inhibitory activity in vitro. However, the antihypertensive effect in vivo is not so evident, and some ACE inhibitory peptides actually have the opposite effect [15,32,33]. Walnut glutelin-1 ACE inhibitory peptides (VERGRRITSV and FVIEPNITPA) showed high inhibitory activity in vitro. However, animal experiments still need to verify whether they also have a blood pressure lowering activity in vivo. The antihypertensive effects of two ACE inhibitory peptides (VERGRRITSV and FVIEPNITPA) in vivo were evaluated by measuring the changes of SBP and DBP.As shown in Figure 5A, the SBP of SHR in each group, before administration, reached approximately 170–180 mmHg. After a single intragastric administration experiment, the SBP of the treatment group decreased rapidly in the first 0–2 h. The maximal reduction in SBP of the captopril-treated, VERGRRITSV-treated (VERGRRITSV-H, and VERGRRITSV-L), and FVIEPNITPA-treated (FVIEPNITPA-H and FVIEPNITPA-L) groups reached 29.5, 32.3, 22.4, 30.4, and 23.0 mmHg, respectively. The SBP of the captopril group and low-dose ACE inhibitory peptide group remained basically stable for 2–8 h. The high-dose ACE inhibitory peptide group showed a slow and continuous decline, while the SBP of the high-dose ACE inhibitory peptide group continued to decrease slowly. Eight hours after administration, the SBP of the high-dose ACE inhibitory peptide groups dropped to approximately 140 mmHg. It can be concluded from the results that the purified ACE inhibitory peptides could reduce SBP after a single administration.

It can be seen from Figure 5B that the SBP of the SHRs negative control group showed an evident increasing trend during the 4-week experiment. After one week of oral administration, the SBP of the captopril positive control group and the ACE inhibitory peptides (VERGRRITSV and FVIEPNITPA) treatment groups were significantly lower than that of the SHRs group (*p* < 0.01). In the first week, the SBP of the VERGRRITSV-L-treated group decreased from 175.3 ± 10.1 mmHg to 137.7 ± 8.2 mmHg; the SBP remained basically stable in the following three weeks.

The DBP of the SHR control group increased rapidly over time, and it was significantly higher than that of the other groups in the second week after administration (*p* < 0.01). On the contrary, the DBP of the captopril, low-dose ACE inhibitory peptide VERGRRITSV, and FVIEPNITPA groups began to decrease after one week of continuous administration, and the decline reached a maximum in the second week. The DBP of the high-dose ACE inhibitory peptide FVIEPNITPA group began to decrease in the second week after administration. Besides, the high-dose ACE inhibitory peptide VERGRRITSV could inhibit the rapid increase in DBP, but its DBP did not decrease (Figure 5C). Our research results clearly show that the ACE inhibitory peptides (VERGRRITSV and FVIEPNITPA) had good effects on reducing the BP level in vivo. Additionally, the low-dose VERGRRITSV group had a better effect than other treatment groups. The results clearly verified that the ACE inhibitory peptides (VERGRRITSV and FVIEPNITPA) of walnut glutelin-1 could significantly reduce blood pressure of SHRs and have great potential in developing functional foods or antihypertensive drugs.

#### 3.4.2. Effects of Glutelin-1 ACE Inhibitory Peptide on Serum ACE, AngII, AGT, and ALD Levels in SHR

**Figure 6:**
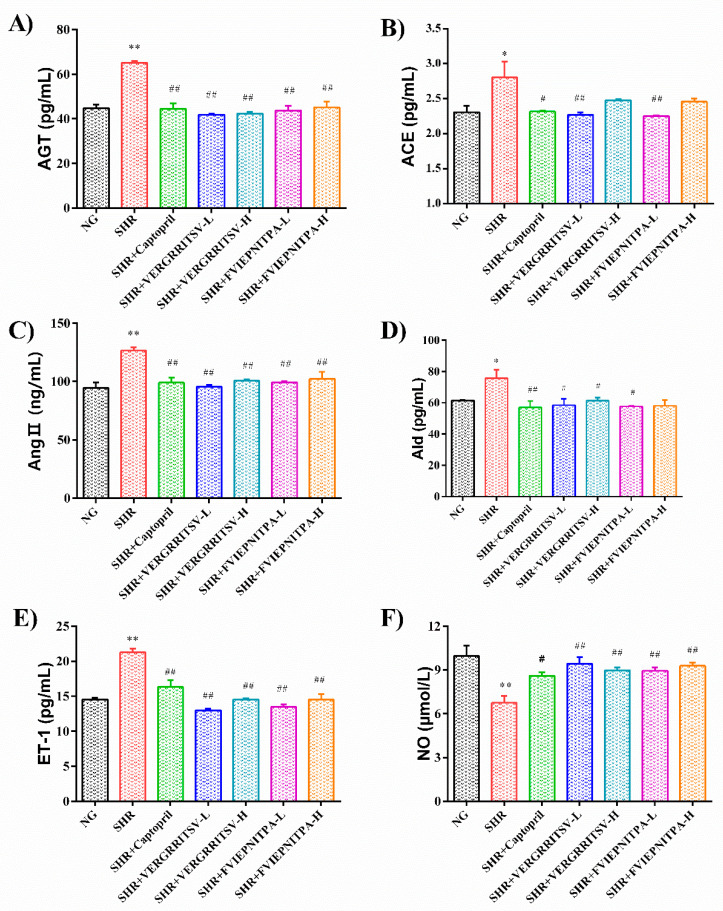
Effects of ACE inhibitory peptides of walnut glutelin-1 on serum AGT (**A**), ACE (**B**), Ang II (**C**), ALD (**D**), ET-1 (**E**), and NO (**F**) levels in SHR. * *p* < 0.05 and ** *p* < 0.01, vs. the normal group (NG). ^#^ *p* < 0.05 and ^##^ *p* < 0.01 compared with the SHRs group.

The renin-angiotensin system (RAS) is a vital body fluid system of the human body. Renin secreted by the parabulbar cells in the glomerulus enters the bulbar artery and can catalyze AGT conversion into angiotensin I (AngI). AngI then produces AngⅡ, which has a solid hypertension-increasing effect under the influence of ACE [34]. AngⅡ can cause the smooth muscle of the blood vessel to contract, increasing the BP level. It also increases ALD secretion, which can boost the reabsorption of sodium in the kidneys’ proximal tubules and cause the retention of sodium and water. As a consequence, the blood sodium concentration and BP levels may surge [10]. As shown in Figure 6A–D, the levels of AGT, ACE, AngⅡ, and ALD in the serum of the SHR control group were significantly higher than those of the captopril-treated, VERGRRITSV-treated, and FVIEPNITPA-treated groups (*p* < 0.05). These findings demonstrated that the walnut glutelin-1 ACE inhibitory peptide could inhibit the ACE activity and over-activated RASS, thereby reducing the production of AngⅡ, preventing the excessive secretion of ALD, slowing the reabsorption of sodium by the kidneys, and lowering BP levels of SHRs.

#### 3.4.3. Effects of the ACE Inhibitory Peptide of Walnut Glutelin-1 on Serum ET-1 and NO Levels in SHR

The balance of NO and ET-1 synthesis and release is the key to maintaining vascular endothelium’s normal function. ET-1 is considered a potent vasoconstrictor secreted by endothelial cells [35]. NO can relax blood vessels, and it has anti-inflammatory, anti-platelet, anti-proliferative, and anti-migration features, playing a central role in the development of endothelial function [36]. The lack of NO produced by endothelial nitric oxide synthase leads to endothelial dysfunction. During hypertension, ACE catalyzes the degradation of bradykinin in the kallikrein system. The dysfunction of the vascular endothelial function leads to an insufficient release of NO synthesis and can increase the release of ET-1 synthesis, resulting in vasoconstriction and surging BP levels [37]. As shown in Figure 6E–F, the content of ET-1 in the serum of the SHR control was significantly higher than that of the other groups (*p* < 0.01), and the NO content was significantly lower than that of the treated groups (*p* < 0.05). In addition, the VERGRRITSV and FVIEPNITPA-treated groups had low ET-1 levels, and the NO content was equivalent to that of the captopril group. Therefore, the walnut glutelin-1 ACE inhibitory peptide could decrease the activity of ACE, slow down the degradation of bradykinin, improve the function of vascular endothelium, and regulate the vasomotor state effectively, consequently lowering the BP level.

### 3.5. Stability of Synthetic Peptides

#### 3.5.1. Effect of In Vitro GI Digestion

**Figure 7:**
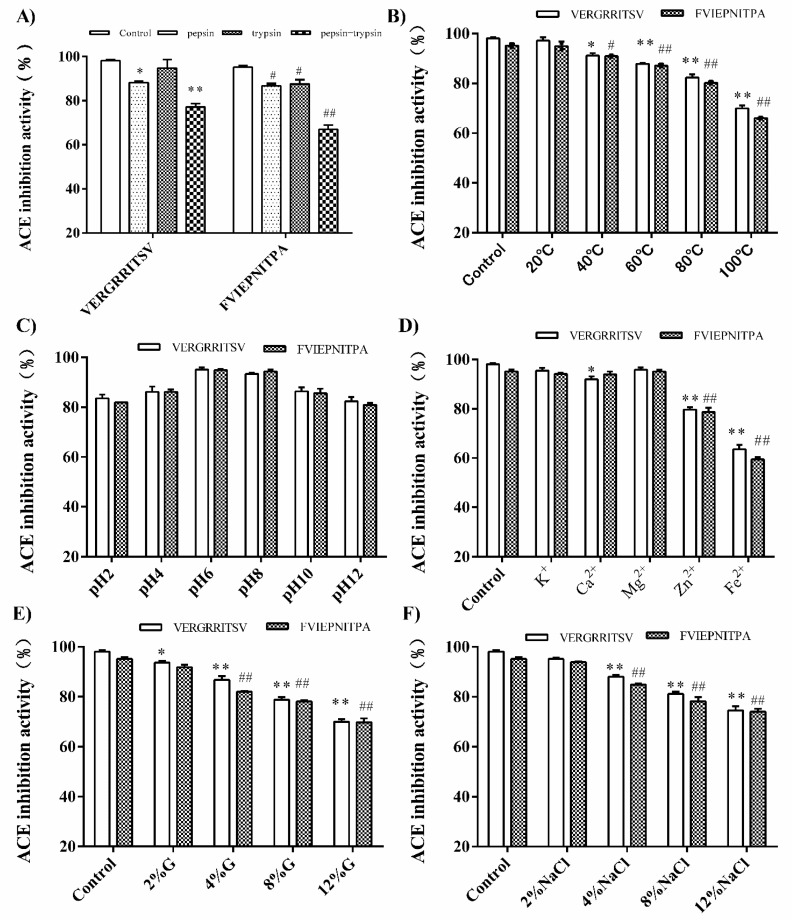
The stability of ACE-inhibitory activity in synthetic peptides VERGRRITSV and FVIEPNITPA exposed to simulated gastrointestinal digestion (**A**). Effect of temperature (**B**), pH (**C**), metal ions (**D**), glucose (**E**), and NaCl (**F**) on the stability of synthetic peptides VERGRRITSV and FVIEPNITPA. * *p* < 0.05 and ** *p* < 0.01, vs. the VERGRRITSV control group. ^#^ *p* < 0.05 and ^##^ *p* < 0.01 compared with the FVIEPNITPA control group.

After entering the human digestive system, the peptides are degraded by digestive enzymes in the GI tract, which have uncertain effects on the spatial structure, amino acid sequence, molecular weight and other aspects of ACE inhibitory peptides [38]. Therefore, some ACE inhibitory peptides with high in vitro activity decreased in vivo activity. However, some other peptides exhibited the opposite behavior [39,40]. It was necessary to verify whether ACE inhibitory peptides can resist the hydrolysis of digestive proteases [41]. The digestibility of the walnut glutelin-1 ACE inhibitor peptide in pepsin and trypsin could be determined by simulating the intestinal digestion environment of gastric juice in vitro. As shown in Figure 7A, the ACE inhibitory peptides (VERGRRITSV and FVIEPNITPA) could still maintain high ACE inhibitory activity under pepsin or trypsin digestion. However, after the combined action of pepsin and trypsin, the ACE inhibitory activity decreased considerably, and the inhibition rate of VERGRRITSV and FVIEPNITPA was reduced to 76.9 and 70.3, respectively. This might be due to the active peptides serving as substrates in the GI tract and were partially hydrolyzed into short peptides, or even amino acids by digestive enzymes, which directly affects the structure of peptides and the ACE inhibitory activity [39,40].

#### 3.5.2. Effect of Temperature, pH, Metal Ion, Sugar, and Salt Treatments

Some environmental factors during food processing, such as temperature, pH, metal ions, salt concentration and sugar concentration, will affect the stability of ACE inhibitory peptides and determine their potential applicability in functional foods [41].The influence of food processing environmental factors on walnut meal glutelin-1 ACE inhibitory peptides (VERGRRITSV and FVIEPNITPA) showed that the ACE inhibitory peptide activity of walnut glutelin-1 did not change much between 20 °C–60 °C (Figure 7B). However, higher temperatures could decrease the ACE inhibitory activity. The ACE inhibition rates remained above 80% at 80 °C and dropped to about 70% at 100 °C, possibly because ACE inhibitory peptides would be degraded and change in structure at higher temperatures [42].

The change of pH value had no significant effect on the activity of walnut glutelin-1 ACE inhibitory peptides (Figure 7C), indicating that the two ACE inhibitory peptides (VERGRRITSV and FVIEPNITPA) had good pH stability. The two peptides could maintain good activity after being hydrolyzed by pepsin, which may be related to their excellent pH stability [43]. Some scholars believed that ACE inhibitory peptides were not easily affected by pH and digestive enzymes, which may be related to their smaller molecular weight [44,45], and our research results may be related to this.

Metal ions directly affected the ACE inhibitory activity of walnut glutelin-1 (Figure 7D). We have observed that ACE inhibitory peptides were very stable in an environment with metal ions such as K^+^, Ca^2+^, and Mg^2+^, whereas such peptides had poor stability under Zn^2+^ and Fe^2+^ conditions. Potassium, calcium, and magnesium are important components of daily food, and walnut glutelin-1 ACE inhibitory peptides can be consumed with foods containing these nutrients without affecting their activity. However, to contain other potential changes in the peptides’ activity, food rich in Zn^2+^ and Fe^2+^, e.g., animal liver, kidney, blood, lean meat, shellfish, kelp, and fungus should be avoided [43,46,47].

Glucose is a common preservative that is widely used in food and health products. It also has varying degrees of influence on the color, taste, and flavor of food. Considering that the Maillard reaction occurs when there are reducing sugars and amino compounds in food, it is necessary to study the effect of glucose on the inhibitory rate of peptides [48]. The ACE inhibitory peptides of the walnut glutelin-1 had high stability under low concentrations of salt; as the concentration increased, the ACE inhibitory activity decreased significantly (Figure 7E). This might be because, as the concentration of glucose increases, the Maillard reaction also becomes stronger, and the glycosidic bond of glucose reacts with the amino group of the peptide, causing the ACE inhibitory peptide to be consumed, and its inhibitory activity to decrease [49].

The results of this study showed that VERGRRITSV and FVIEPNITPA had good stability under low-concentration salt conditions, but they became less stable under high-concentration salt conditions (Figure 7F). This is because the structure of walnut glutelin-1 ACE inhibitory peptide may change under high salt conditions, and the side chains of amino acids may be damaged by NaCl treatment, which reduces the ACE inhibitory activity [50].

The ACE inhibitory peptides (VERGRRITSV and FVIEPNITPA) isolated from the hydrolysate of walnut glutelin-1 showed stability across a range of pH values, at temperatures < 60 °C, and in the presence of metal ions (K^+^, Ca^2+^, and Mg^2+^) under low concentration of salt and glucose. These results indicated that the activity of ACE inhibitory peptides (VERGRRITSV and FVIEPNITPA) would not be greatly affected by appropriate processing conditions, which suggested the possible therapeutic use of these peptides as antihypertensive agents in vivo.

## 4. Conclusions

In this study, two novel peptides (VERGRRITSV and FVIEPDITPA) were successfully isolated and identified from walnut glutelin-1 hydrolysate. The results of animal experiments showed that the oral administration of the two ACE inhibitory peptides could significantly decrease systolic and diastolic blood pressure in SHRs. On the one hand, the two ACE inhibitory peptides could reduce the AGT, ACE, Ang II, and ALD levels in SHRs by regulating the RAS system. On the other hand, the level of ET-1 decreased, whereas the level of NO in serum increased by regulating the kinin system, lowering the BP level as a consequence. These results suggested that the two ACE inhibitory peptides have the potential to treat hypertension. Moreover, the molecular docking study revealed that the ACE inhibition of VERGRRITSV and FVIEPNITPA were mainly attributed to forming very strong hydrogen bonds with the active pockets of ACE. In addition, the two ACE inhibitory peptides displayed good resistance to digestive enzymes, and they could maintain structural stability in ordinary food processing environments. We have extensively verified that the walnut meal glutelin-1 ACE inhibitory peptides (VERGRRITSV and FVIEPNITPA) exhibited significant ACE inhibitory activity in vitro and in vivo, and concluded that this is a valuable feature to develop antihypertensive drugs and food. Meanwhile, walnut meal may have a potential active ingredient with antihypertensive properties, and its development and utilization as an antihypertensive agent will improve the value of this underutilized protein resource. However, the clinical application of ACE inhibitory peptides is still very limited, and further studies on the clinical efficacy and bioavailability of these peptides in humans needs to be further investigated.

## Figures and Tables

**Table 1:** Amino acid sequences, molecular weights, and IC 50 values on angiotensin converting enzyme (ACE) of walnut glutelin-1 hydrolysate ACE inhibitory peptides.

Fraction	Retention Time (Min)	Amino Acid Sequence	Mass (Da)	IC_50_ (μM)
P5	16.120	Val-Glu-Arg-Gly-Arg-Arg-Ile-Thr-Ser-Val (VERGRRITSV)	1172.35	6.82
P8	17.635	Phe-Val-Ile-Glu-Pro-Asn-Ile-Thr-Pro-Ala (FVIEPNITPA)	1100.28	6.36

## Data Availability

All data presented in this study are available on request from the corresponding author. The data are not uploaded in publicly accessible databases.
